# Two New Flavonoids from the Leaves of *Baccharis oblongifolia* (Ruiz and Pav.) Pers. (Asteraceae)

**DOI:** 10.3390/molecules24173198

**Published:** 2019-09-03

**Authors:** Paulo R. F. Zampieri, Cinthia I. Tamayose, Oriana A. Fávero, Paulete Romoff, Marcelo J. P. Ferreira

**Affiliations:** 1 Departamento de Botânica, Instituto de Biociências, Universidade de São Paulo, São Paulo 05508-090, Brazil; 2 Universidade Presbiteriana Mackenzie, São Paulo CEP 01302-907, Brazil

**Keywords:** *Baccharis*, Astereae, Compositae, flavonoids, chlorogenic acid derivatives, antiradical properties

## Abstract

In this work, two new flavonoids, oblongifolioside A (**1**) and oblongifolioside B (**2**), along with eight known compounds (**3**–**10**), are isolated from the leaves of *Baccharis oblongifolia* (Asteraceae). The new structures are established through spectroscopic data and the known compounds are identified by comparison with data reported in the literature. The compounds (**1**–**10**) are evaluated in relation to their antiradical properties. Compounds **1** and **2** are found to exhibit high antiradical activity compared to their respective non-acylated flavonoids.

## 1. Introduction

*Baccharis* L. (Astereae, Asteraceae) is a large New World genus comprising between 354 and 500 species [1,2]. Approximately 90% of *Baccharis* species are found in South America and distributed mainly in the warm temperate and tropical regions of Argentina, Brazil, Chile, Colombia, and Mexico. In Brazil, 179 species are found chiefly in the Southern and Southeastern regions. Among them, 115 are endemic species [3] which are restricted to the elevated altitudes of the Atlantic Forest.

Some *Baccharis* species are used in folk medicine, including for spasmolytic, diuretic, and analgesic purposes and for the treatment of ulcers, fever, gastrointestinal illnesses, diabetes, and microbial infections [4,5,6]. Previous phytochemical studies of this genus have reported several classes of natural products, such as chlorogenic acid derivatives [4,7,8,9,10], terpenoids [11,12,13,14,15,16,17,18,19,20,21,22], especially diterpenes with dozens of isolated compounds from *Baccharis* [4,5,17,18,19,20,21,22], and flavonoids from flavone, flavanone, and flavonol types [4,5,21,22,23,24,25,26]. Furthermore, it has also been described as having diverse biological activities including antimicrobial [4,5,6,14,15], antiviral [4,5,6], antiprotozoal [9,24,25], anti-inflammatory [10,13], cytotoxic [4,5,6,23], and antioxidant [7,13,14,15,22,23], as well as antimutagenic and chemopreventive effects [4,5,6].

**Figure 1:**
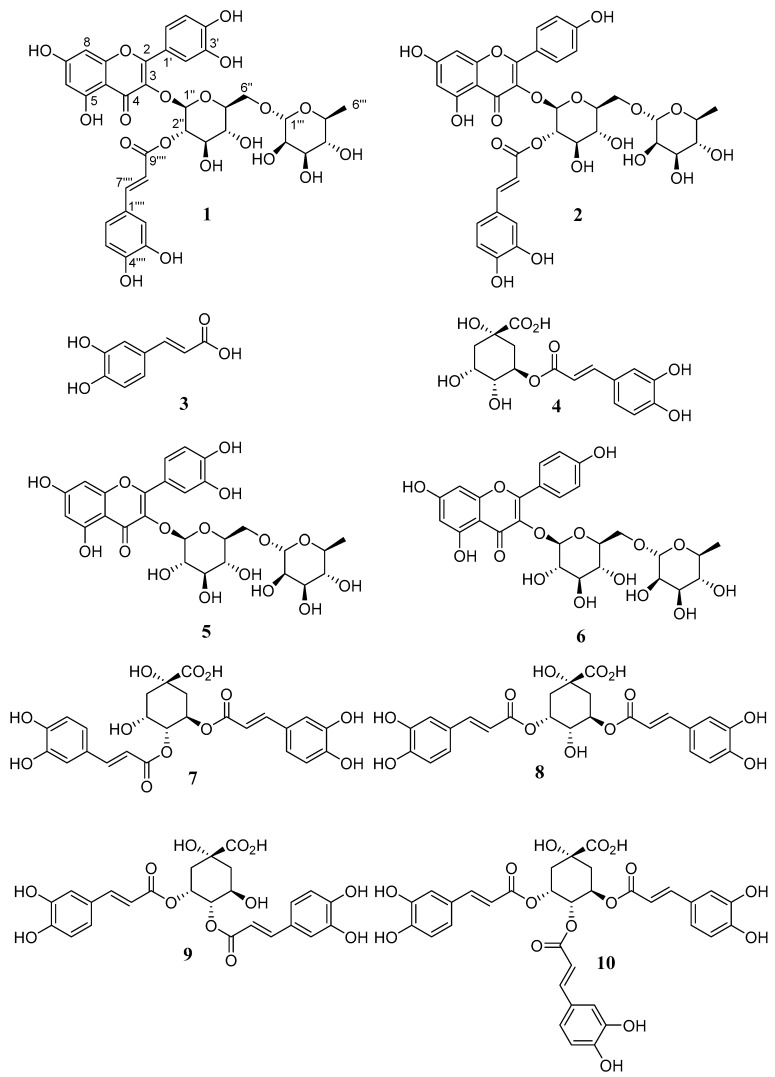
Compounds identified from *B. oblongifolia* leaves.

In our continuing efforts in the search for new bioactive compounds from Brazilian Asteraceae species, this paper describes two new acylated flavonoids, oblongifolioside A (**1**) and oblongifolioside B (**2**), and eight known compounds (**3**–**10**) from *B. oblongifolia* leaves, as shown in Figure 1. The new structures were elucidated by spectroscopic data and the known ones were identified by comparison with literature data. Herein, we report the isolation, structure elucidation, and antiradical activities of these compounds.

## 2. Results

### Structural Elucidation

Oblongifolioside A (**1**) was isolated as a yellow powder. The molecular formula of **1** was assigned C_36_H_36_O_19_ based on its HRESIMS at *m/z* 771.1772 [M − H]^−^, indicating 19 degrees of unsaturation. The UV spectrum exhibited absorption maxima at 254 and 345 nm, with a shoulder at 315 nm. The ^1^H NMR spectrum showed four doublets at δ 6.29 (1H, *J* = 2.0 Hz, H-6) and δ 6.13 (1H, *J* = 2.0 Hz, H-8) referring to the *meta*-coupling of H-6 and H-8 hydrogens from an A-ring of flavonoids, and δ 7.57 (1H, *J* = 2.1 Hz, H-2′), δ 6.86 (1H, *J* = 8.1 Hz, H-5′) and a doublet of doublets at δ 7.56 (1H, *J* = 2.1 Hz and 8.1 Hz) attributed to a 1′,3′,4′-trisubstituted B-ring of flavonoids. A quercetin derivative was suggested by the absence of additional signals pertaining to hydrogen at H-3 as well as methoxyl groups. The aromatic region also showed two other doublets at 7.62 (1H, *J* = 15.8 Hz, H-7⁗) and δ 6.33 (1H, *J* = 15.8 Hz, H-8⁗) assigned to *trans*-hydrogens from an α,β-unsaturated carboxyl group, two doublets at δ 7.06 (1H, *J* = 2.1 Hz, H-2⁗) and δ 6.78 (1H, *J* = 8.2 Hz, H-5⁗) corresponding to *meta*- and *ortho*-couplings between hydrogens, respectively, and a doublet of doublets at δ 6.94 (1H, *J* = 2.1 Hz and 8.2 Hz, H-6⁗). These data allowed for the verification of the presence of a caffeoyl group in the structure. Additionally, the ^1^H NMR spectrum showed a doublet at δ 5.48 (1H, *J* = 8.0 Hz, H-1″) and a broad singlet at δ 4.55 (1H, H-1‴), which is characteristic of hydrogens bonded in anomeric carbons. Three additional doublets at 3.87 (1H, *J* = 9.5 Hz, H-6″), δ 3.27 (1H, *J* = 9.5 Hz, H-6″) and δ 1.13 (3H, *J* = 6.2 Hz, H-6‴) suggested the presence of a glycosyl moiety substituted at C-6″ and a rhamnosyl unit in the structure. The doublet of doublets at δ 5.04 (1H, *J* = 8.0 Hz and 9.6 Hz, H-2″) suggested the presence of a substituent group at C-2″ from the glycosyl moiety (Figures S1 and S2).

**Figure 2:**
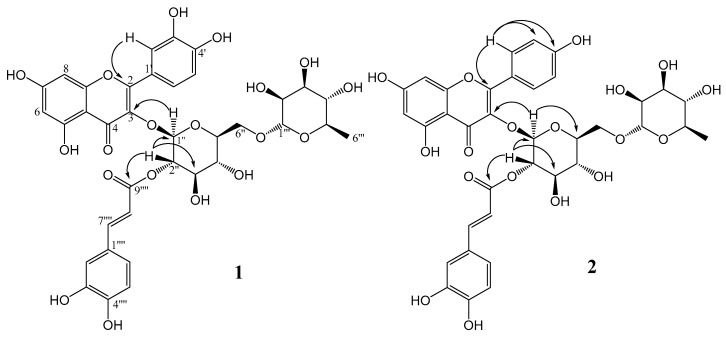
Key HMBC correlations of compounds **1** and **2**.

The ^13^C NMR spectrum displayed thirty-six carbon signals, including a carbonyl, a carboxyl, twelve quaternary carbons, ten unsaturated methines, ten oxymethines, one oxygenated methylene, and one methyl group. Through HMBC correlations, it was possible to establish the connectivity among groups in the structure. The signals at δ 5.48 and δ 5.04 attributed to H-1″ and H-2″ from the glycosyl moiety correlated with the carbons at δ 133.3 and δ 167.2, respectively, which were attributed to C-3 from quercetin and to C-9⁗ from a caffeoyl group. These correlations confirmed the glucose at C-3 of quercetin and the caffeoyl group esterified on hydroxyl at C-2″ of glucose. Other key HMBC correlations are shown in Figure 2. With the aid of HSQC and HMBC experiments, all ^1^H and ^13^C NMR signals of **1** were assigned as shown in Table 1 (Figure S3–S5).

Oblongifolioside B (**2**) was isolated as a yellow powder. The molecular formula of **2** was assigned as C_36_H_36_O_18_ based on its HRESIMS at *m/z* 755.1881 [M − H]^−^, indicating 19 degrees of unsaturation. The UV spectrum exhibited absorption maxima at 260 and 330 nm, with a shoulder at 300 nm. The ^1^H NMR spectrum showed two doublets at δ 7.99 (2H, *J* = 8.4 Hz, H-2′,6′) and δ 6.90 (2H, *J* = 8.4 Hz, H-3′,5′) attributed to *ortho*-coupling of hydrogens from a *para*-substituted B-ring of flavonoids. Two broad singlets were observed at δ 6.35 (1H, H-8) and δ 6.17 (1H, H-6) assigned to hydrogens from an A-ring of flavonoids. A kaempferol derivative was suggested by the absence of additional signals pertaining to hydrogen at H-3 as well as methoxyl groups (Figure S6–S11).

Similarly to structure 1, the ^1^H NMR spectrum showed two other doublets at 7.62 (1H, *J* =15.8 Hz, H-7⁗) and δ 6.32 (1H, *J* =15.8 Hz, H-8⁗) assigned to *trans*-hydrogens from an α,β-unsaturated carboxyl group, two doublets at δ 7.06 (1H, *J* = 2.0 Hz, H-2⁗) and δ 6.79 (1H, *J* = 8.2 Hz, H-5⁗), and a doublet of doublets at δ 6.95 (1H, *J* = 2.0 Hz and 8.2 Hz, H-6⁗) assigned to the presence of a caffeoyl group in the structure. Additionally, the spectrum showed two doublets at δ 5.55 (1H, *J* = 8.0 Hz, H-1″) and δ 4.54 (1H, *J* = 1.4 Hz, H-1‴), which is typical of hydrogens bonded in anomeric carbons. Three additional doublets at 3.87 (1H, *J* = 9.3 Hz, H-6″), δ 3.43 (1H, *J* = 9.3 Hz, H-6″) and δ 1.13 (3H, *J* = 6.2 Hz, H-6‴) suggested the presence of a glycosyl moiety substituted at C-6″ and a rhamnosyl unit in the structure. The doublet of doublets at δ 5.01 (1H, *J* = 8.0 Hz and 9.6 Hz, H-2″) suggested the presence of a substituent group at C-2″ from a glycosyl moiety.

The ^13^C-NMR spectrum displayed thirty-four carbon signals, including a carbonyl, a carboxyl, twelve quaternary carbons, eight unsaturated methines, ten oxymethines, one oxygenated methylene, and one methyl group. Through HMBC correlations, it was possible to establish the connectivity among groups in the structure. The signals at δ 5.55 and δ 5.01 attributed to H-1″ and H-2″ of the glycosyl moiety correlated with the carbons at δ 133.3 and δ 167.1, respectively, which were attributed to C-3 from kaempferol and to C-9⁗ from a caffeoyl group, confirming the bond of glucose at C-3 of kaempferol and the caffeoyl group esterified on hydroxyl at C-2″ of glucose. Other key HMBC correlations are shown in Figure 2 and other signals of compound **2** were assigned and are shown in Table 1.

The known compounds were identified as caffeic acid (**3**) [27], chlorogenic acid (5-*O*-(*E*)-caffeoylquinic acid (**4**)) [28], rutin (quercetin-3-*O*-rutinoside (**5**)) [29], nicotiflorin (kaempferol-3-*O*-rutinoside (**6**)) [30], 4,5-di-*O*-(*E*)-caffeoylquinic acid (**7**) [31], 3,5-di-*O*-(*E*)-caffeoylquinic acid (**8**) [31], 3,4-di-*O*-(*E*)-caffeoylquinic acid (**9**) [31], and 3,4,5-tri-*O*-(*E*)-caffeoylquinic acid (**10**) [28] by analyzing their spectral data and comparing them with reported literature values.

Flavonoids and chlorogenic acid derivatives are components known for their antiradical activity [30]. Thus, the isolated compounds (**1**–**10**) were tested for their antiradical activity using a DPPH assay. Compounds **1** and **2** exhibited a high activity compared to Trolox (Table 2). These components had higher antiradical activity than their respective non-acylated flavonoids. All chlorogenic acids were more active than Trolox as observed previously in the literature [31]. Additionally, the chlorogenic acid derivatives substituted by caffeoyl groups at the C-4 position showed antiradical activity compatible with the new acylated flavonoids.

## 3. Materials and Methods

### 3.1. General Experimental Procedures

Column chromatography (CC) was performed on a Sephadex LH-20 (GE Healthcare). HPLC grade solvents of the trademark T.J. Baker were used for the HPLC chromatography analyses. Analytical HPLC analyses were carried out on an Agilent 1260 system (G1311 pump 110 and G1315D photodiode array detector; Palo Alto, US) with a 60 mm flow cell. Zorbax Eclipse plus reverse phase C18 (4.6 mm × 150 mm, 3.5 μm, Agilent) was used as the stationary phase and a flow rate of 1.0 mL·min^−1^ was employed for analyses on the analytical scale. For separation of compounds the Agilent 1200 semi-preparative chromatograph system (Palo Alto, US) was used with a C18 Zorbax eclipse plus LC-18 column (25 cm × 10 mm) with 5 μm diameter particles and a flow rate of 4.176 mL·min^−1^ of solvent A: milli-Q water acidified with 0.1% acetic acid (*v/v*) and solvent B: acetonitrile (ACN). The column temperature was 45°C, the injection volume of the sample was 200 μL and the sample was dissolved in methanol at a concentration of 100 g·L^−1^. NMR spectra of hydrogen-1 (^1^H-NMR) and carbon-13 (^13^C-NMR) were recorded on a Bruker AIII 500 MHz spectrometer (MA, US) operating at 500 MHz for ¹H-NMR and 125 MHz for ¹³C-NMR at the Institute of Chemistry of the University of São Paulo. The spectra were obtained in deuterated methanol from Sigma-Aldrich as a solvent. NMR data were processed using MestreNova 9.0 software. Optical rotations were measured on a Perkin Elmer 243B polarimeter. Mass spectra were recorded on an Amazon ETD Bruker Daltonics (MA, US) with capillary 4500V and nebulizer at 27 psi in a negative mode.

### 3.2. Plant Material

Leaves of *Baccharis oblongifolia* (Ruiz and Pav.) Pers. (Asteraceae) were collected from Campos do Jordão, São Paulo, Brazil on October 22, 2016 (−22°45′47″ S; −45°35′02″ W). The plant was identified by Professor Dr. Oriana A. Fávero and a voucher specimen (Fávero et al. 535) was deposited at the Herbarium of the University of São Paulo (SPF).

### 3.3. Extraction and Isolation

Dried leaves of *B. oblongifolia* (373 g) were powdered and extracted with hexane and subsequently with ethanol (EtOH). EtOH extract (79.8 g) was resuspended in EtOH:H_2_O (1:1) and partitioned successively with hexanes, dichloromethane (DCM), and ethyl acetate (EtOAc).

The EtOAc phase (4.5 g) was subjected to Sephadex LH-20 column chromatography and eluted with methanol to afford seven groups (Gr. A–G). Gr. B and C were subjected to separation by semi-preparative HPLC (0–3 min: 10 → 20% ACN; 3–7 min: 20% ACN) to afford, respectively, caffeic acid (**3**, 45.3 mg) and chlorogenic acid (**4**, 90.5 mg). Gr. D was subjected to semi-preparative HPLC (0–3 min: 10 → 20% ACN; 3–7 min: 20% ACN; 7–8 min: 20 → 25% ACN; 8–12 min: 25% ACN) to give rutin (**5**, 22.4 mg, quercetin-3-*O*-rutinoside) and nicotiflorin (**6**, 11.5 mg, kaempferol-3-*O*- rutinoside). Gr. E was subjected to semi-preparative HPLC (0–3 min: 10 → 20% ACN; 3–7 min: 20% ACN; 7–8 min: 20 → 25% ACN; 8–12 min: 25% ACN; 12–17 min: 25 → 50% ACN; 17–22 min: 50 → 100% ACN; 22–22.5 min: 100% ACN) to yield 4,5-di-*O*-(*E*)-caffeoylquinic acid (**7**, 77.3 mg), 3,5-di-*O*-(*E*)-caffeoylquinic acid (**8**, 235.9 mg), and 3,4-di-*O*-(*E*)-caffeoylquinic acid (**9**, 95.5 mg). The same elution conditions for Gr. E were used to subject Gr. F and G to semi-preparative HPLC. Gr. F afforded oblongifolioside A (**1**, 27.8 mg, quercetin-3-*O*-β-[2″-*O*-(*E*)-caffeoyl]-rutinoside) and oblongifolioside B (**2**, 16.8 mg, kaempferol-3-*O*-β-[2″-*O*-(*E*)-caffeoyl]-rutinoside) while Gr. G yielded 3,4,5-tri-*O*-(*E*)- caffeoylquinic acid (**10**, 5.0 mg).

Oblongifolioside A (**1**): yellow powder; [α]^25^_D_ +11.7° (c 0.01, MeOH). ^1^H and ^13^C-NMR spectral data, see Table 1; HR-ESI-MS: *m/z* [M − H]^−^, calcd. for C_36_H_35_O_19_: 771.1772, found: 771.1711.

Oblongifolioside B (**2**): yellow powder; [α]^25^_D_ +13.4° (c 0.01, MeOH). ^1^H and ^13^C-NMR spectral data, see Table 1; HR-ESI-MS: *m/z* [M − H]^−^, calcd. for C_36_H_35_O_18_: 755.1823, found: 755.1881.

### 3.4. Antiradical Assay

Evaluation of antiradical activity was performed according to a protocol published in the literature [32]. Briefly, the DPPH solution was prepared from 3.5 mg to 3.9 mg of DPPH in 50 mL of methanol. The exact concentration of the DPPH solution was determined spectrophotometrically by the maximum absorbance at 515 nm (ε_DPPH_ = 1.25 × 10^4^ L·mol^−1^·cm^−1^). The Trolox antiradical solution was prepared with 1.25 mg in 2.5 mL methanol. The solutions prepared remained for 5 min in a sonicator for complete solubilization.

Analyses were performed on a microplate reader for absorbance with an optical path of 5 mm with a total volume of 220 μL. Measurements were initiated by the addition of 200 μL DPPH in 20 μL of the sample solution (pure compound). The kinetics of the reactions were observed by absorbance of the DPPH solution at 515 nm. All kinetic tests were performed in triplicate in independent measurements and the results treated and represented with mean ± standard deviation in the program Origin Pro 8.5 to obtain the kinetic curves.

The variation of absorbance (ΔAbs.) between T0 and T50 (AbsTinitial-AbsTfinal) shows a linear correlation with the antiradical concentration. In order to calculate the antiradical activity of the phases and pure compounds, the angular coefficients (α) of the antiradical (A) and Trolox (T) standard deviations were used as a function of the absorbance variation in order to obtain the corresponding antiradical capacity as a percentage of Trolox (%Tx), according to Equation (1) [32]:

%Tx = (αA/αT) × 100(1)

In addition to correlating the concentration of the antiradical compound with the variation of the absorbance, the correlation of this concentration was also made directly with the concentration of the DPPH radical consumed. From this correlation it was possible to directly obtain the number of DPPH radicals sequestered per antiradical molecule.

The percentage of antiradical activity was calculated from Equation (2), where the negative control was prepared with 200 μL of DPPH and 20 μL of methanol, the blank was prepared with 20 μL of the sample and 200 μL of methanol, and the sample was prepared with 20 μL of the sample and 200 μL of DPPH. The 50% inhibitory concentration (IC_50_) of each compound was obtained from the equation of the straight line of the concentration graph by the percentage of antiradical activity.

AA% = 100 − {[(ABS_SAMPLE_ − ABS_BLANK_) × 100]/ABS_NEGATIVE_} (2)

## Figures and Tables

**Table 1:** ^1^H, ^13^C and HMBC NMR data of **1** and **2** (δ in ppm, *J* in Hz, CD_3_OD).

Position	1			2		
	δ_H_	δ_C_	HMBC	δ_H_	δ_C_	HMBC
2	-----	157.4	-----	-----	157.5	-----
3	-----	133.4	-----	-----	133.3	-----
4	-----	177.6	-----	-----	177.7	-----
5	-----	161.7*	-----	-----	161.7	-----
6	6.13 d (2.0)	99.5	C_5_; C_7_; C_8_; C_10_	6.17 brs	98.5	C_5_; C_7_; C_8_; C_10_
7	-----	165.2*	-----	-----	164.5	-----
8	6.29 d (2.0)	93.8*	C_6_	6.35 brs	93.5	C_6_; C_1⁗_; C_9⁗_
9	-----	157.0	-----	-----	157.0	-----
10	-----	103.4	-----	-----	104.4	-----
1′	-----	121.5	-----	-----	121.5	-----
2′	7.57 d (2.1)	122.1	C_2_; C_6′_; C_4′_	7.99 d (8.4)	130.8	C_2_; C_2′/6′_; C_3′/5′_; C_4′_
3′	-----	144.5	-----	6.90 d (8.4)	114.8	C_1′_; C_3′/5′_; C_4′_
4′	-----	148.4	-----	-----	160.0	-----
5′	6.86 d (8.2)	114.8	C_1′_; C_3′_; C_4′_	6.90 d (8.4)	114.8	C_1′_; C_3′/5′_; C_4′_
6′	7.56 dd (8.2, 2.1)	115.9	C_4′_; C_5′_	7.99 d (8.4)	130.8	C_2_; C_2′/6′_; C_3′/5′_; C_4′_
1″	5.48 d (8.0)	99.5	C_3_	5.55 d (8.0)	99.4	C_3_; C_5″_
2″	5.04 dd (9.6, 8.0)	74.3	C_1″_; C_3″_; C_9⁗_	5.01 dd (9.6, 8.0)	74.3	C_1″_; C_3″_; C_9⁗_
3″	3.60 d (9.6)	74.9	C_2″_; C_4″_	3.61 d (9.6)	74.8	C_2″_; C_4″_
4″	3.43 d (4.7)	70.7	C_5″_; C_6″_	3.35 d (2.4)	70.4	C_5″_; C_6″_
5″	3.38 d (8.5)	75.8	C_4″_; C_6″_	3.43 d (9.3)	76.0	C_4″_; C_6″_
6″	3.87 d (9.5) 3.55 d (9.5)	66.9	C_5″_; C_1‴_	3.87 d (9.3) 3.43 d (9.3)	66.9	C_4″_; C_1‴_
1‴	4.55 brs	100.9	C_3‴_; C_5‴_	4.54 d (1.4)	100.9	C_3‴_; C_5‴_
2‴	3.49 d (3.1)	70.7		3.64 d (3.3)	70.7	
3‴	3.66 dd (3.3, 1.7)	70.9	C_4‴_	3.53 dd (9.5, 3.3)	70.9	C_4‴_
4‴	3.27 d (9.5)	72.5	C_3‴_; C_5‴_; C_6‴_	3.27 d (9.5)	72.5	C_3‴_; C_5‴_; C_6‴_
5‴	3.46 d (6.4)	68.4	C_4‴_	3.47 dd (9.5, 6.2)	68.4	C_4‴_
6‴	1.13 d (6.2)	16.5	C_4‴_; C_5‴_	1.13 d (6.2)	16.5	C_4‴_; C_5‴_
1⁗	-----	126.5	-----	-----	126.5	-----
2⁗	7.06 d (2.1)	113.8	C_3⁗_; C_4⁗_; C_6⁗_	7.06 d (2.0)	113.8	C_3⁗_; C_4⁗_; C_6⁗_
3⁗	-----	145.4	-----	-----	145.5	-----
4⁗	-----	148.2	-----	-----	148.2	-----
5⁗	6.78 d (8.2)	115.1	C_1⁗_; C_3⁗_; C_4⁗_; C_6⁗_	6.79 d (8.2)	115.1	C_1⁗_; C_3⁗_; C_4⁗_; C_6⁗_
6⁗	6.94 dd (8.2, 2.1)	121.7	C_4⁗_; C_7⁗_; C_8⁗_	6.95 dd (8.2, 2.0)	121.7	C_4⁗_; C_7⁗_; C_8⁗_
7⁗	7.62 d (15.8)	146.0	C_1⁗_; C_2⁗_; C_6⁗_; C_9⁗_	7.62 d (15.8)	146.0	C_1⁗_; C_2⁗_; C_6⁗;_ C_9⁗_
8⁗	6.33 d (15.8)	113.4	C_1⁗_; C_9⁗_	6.32 d (15.8)	113.8	C_1⁗_; C_9⁗_
9⁗	-----	167.2	-----	-----	167.1	-----

Legend: d, doublet; dd, doublet of doublets; brs, broad singlet; *, chemical shift obtained from HSQC and HMBC spectra.

**Table 2:** Antiradical activity of compounds **1**–**2**.

Compound	% Trolox (L·μmol^−1^)	IC_50_ (µmol·L^−1^)	n *
(**1**) Oblongifolioside A	245.5 ± 0.1	13.3 ± 0.5	4.8 ± 0.1
(**2**) Oblongifolioside B	244.9 ± 0.2	12.4 ± 0.2	4.8 ± 0.2
Trolox	--	45.4 ± 1.7	1.96 ± 0.06

Legend: n *, number of radicals trapped by molecule of antiradical.

## Supplementary Materials

The following are available online. Figures S1–S11: Spectral data of compounds 1 and 2.
