# Supplementary material for: Two New Flavonoids from the Leaves of Baccharis oblongifolia (Ruiz and Pav.) Pers. (Asteraceae)
Source: Molecules. 2019 Sep 3;24(17):3198. doi: 10.3390/molecules24173198 (PMC6749570; doi:10.3390/molecules24173198)
Supplement: Supplementary file 1 [file molecules-24-03198-s001.pdf]

SUPPORTING INFORMATION

Two new flavonoids from the leaves of *Baccharis oblongifolia* (Ruiz & Pav.) Pers. (Asteraceae)

Paulo R. F. Zampieri [1], Cinthia I. Tamayose [1], Oriana A. Fávero [2], Paulete Romoff [2], and Marcelo J. P. Ferreira [1,*]

[1] Departamento de Botânica, Instituto de Biociências, Universidade de São Paulo, 05508-090, São Paulo, Brazil; paulorfzampieri@usp.br (P.R.F.Z.); cinthiatamay@gmail.com (C.I.T.); marcelopena@ib.usp.br (M.J.P.F.)

[2] Universidade Presbiteriana Mackenzie, CEP 01302-907, São Paulo, Brazil; oriana.favero@mackenzie.br (O.A.F.); pauleteromoff@yahoo.com.br (P.R.)

* Correspondence: marcelopena@ib.usp.br; Tel.: +55-11-3091-7546 (M.J.P.F.)

List of Supporting Information

Figures S1–5. Spectral data of compound 1: oblongifolioside A [quercetin-3-O-β-[2′′-O-(*E*)-caffeoyl]-rutinoside

Figures S6-11. Spectral data of compound 2: oblongifolioside B [kaempferol-3-O-β-[2′′-O-(*E*)-caffeoyl]-rutinoside

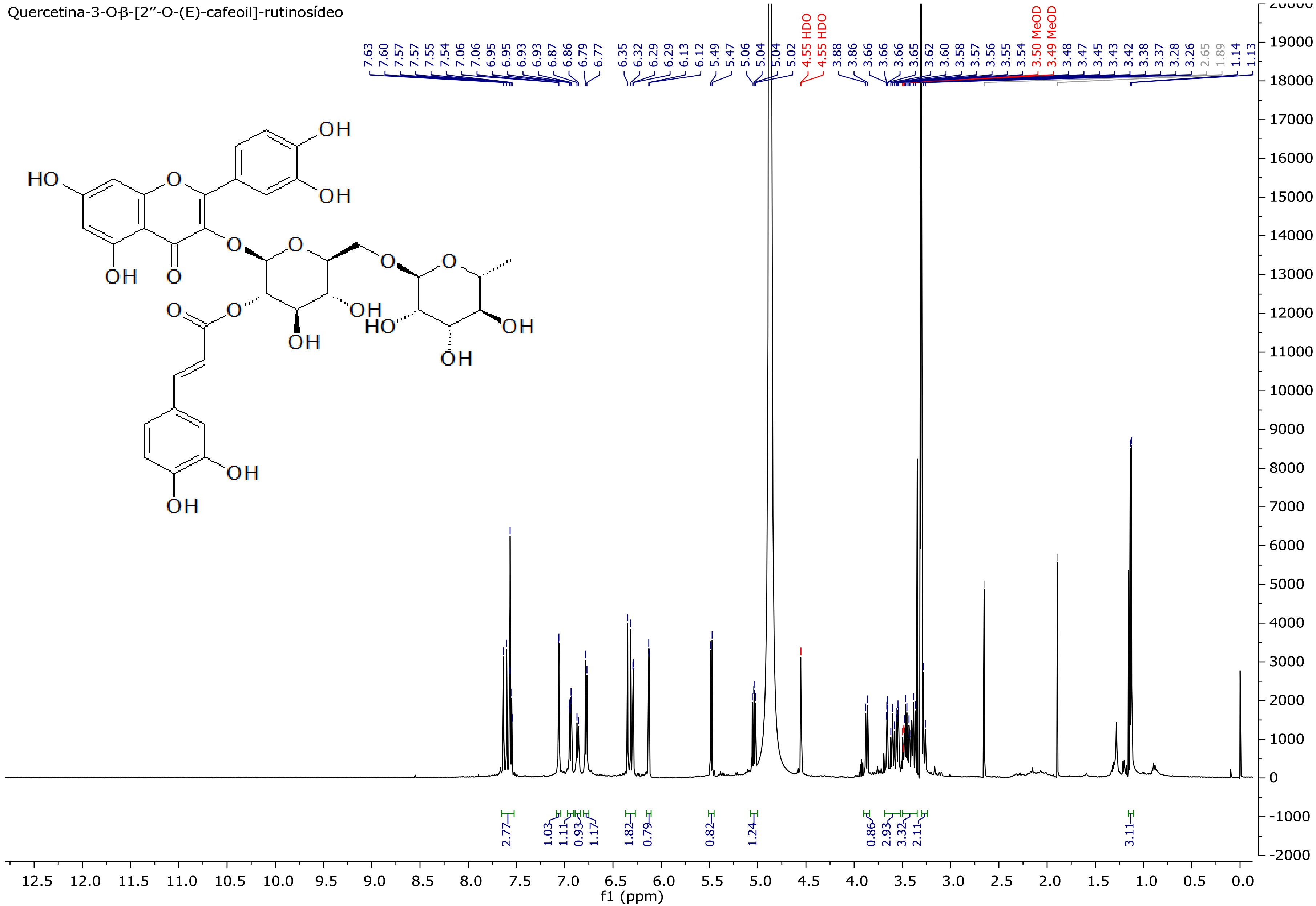

Figure S1. ^{1}H NMR spectrum of compound 1: oblongifolioside A [quercetin-3-O-β-[2’’-O-(*E*)-caffeoyl]-rutinoside

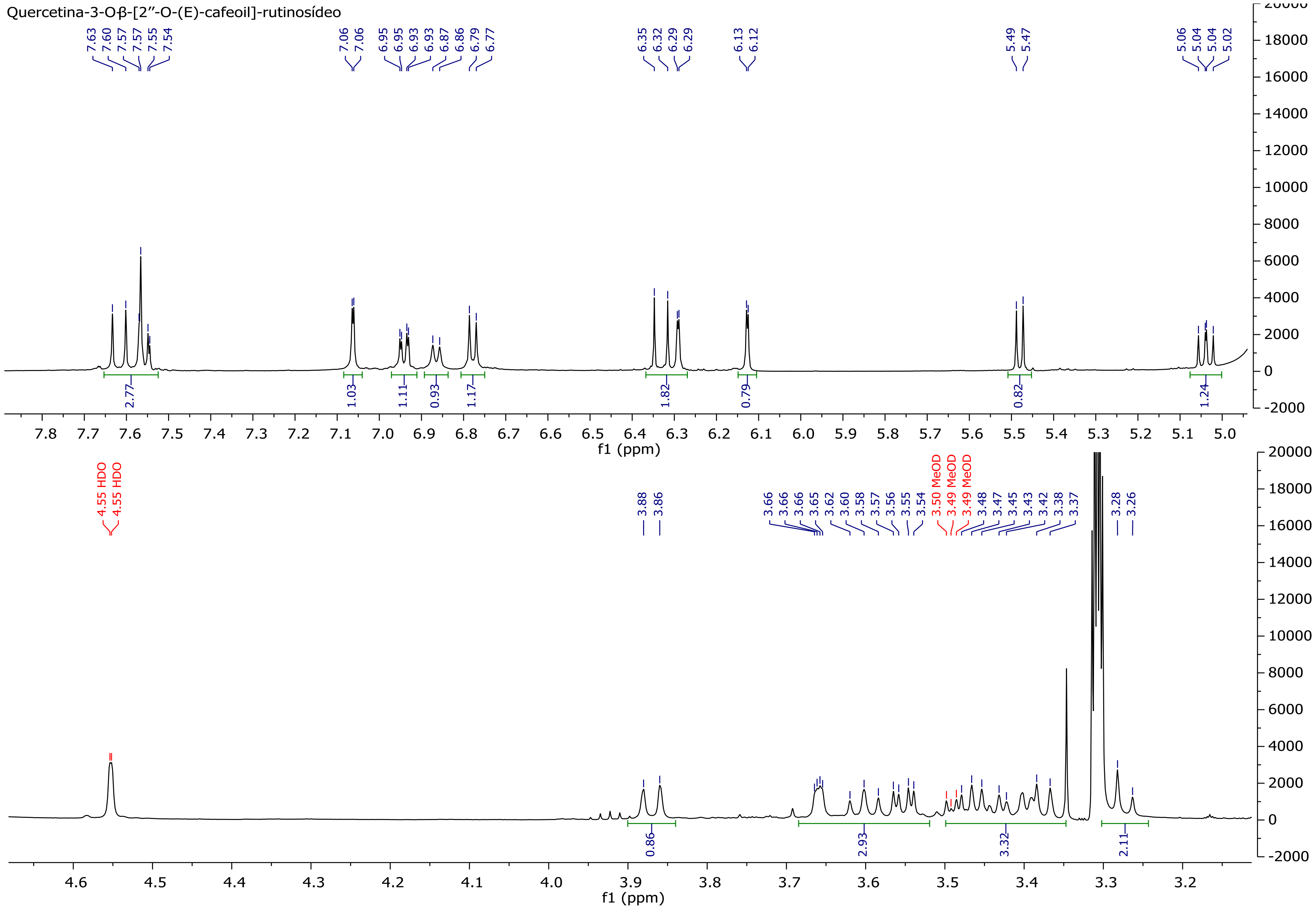

Figure S2. Expansion of ^{1}H NMR of compound 1: oblongifolioside A [quercetin-3-O-β-[2''-O-(*E*)-caffeoyl]-rutinoside

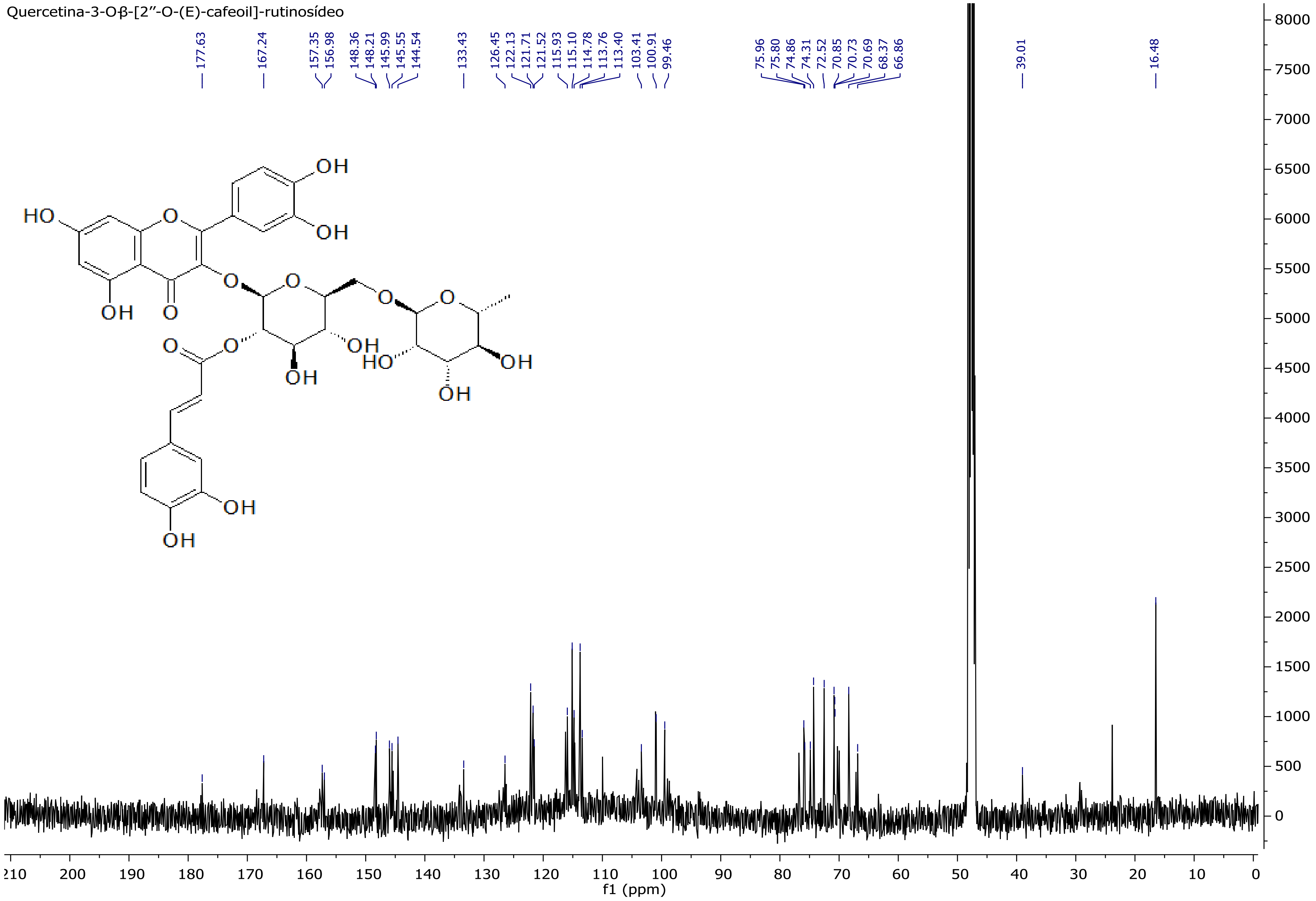

Figure S3. ^{13}C NMR spectrum of compound 1: oblongifolioside A [quercetin-3-O-β-[2''-O-(*E*)-caffeoyl]-rutinoside

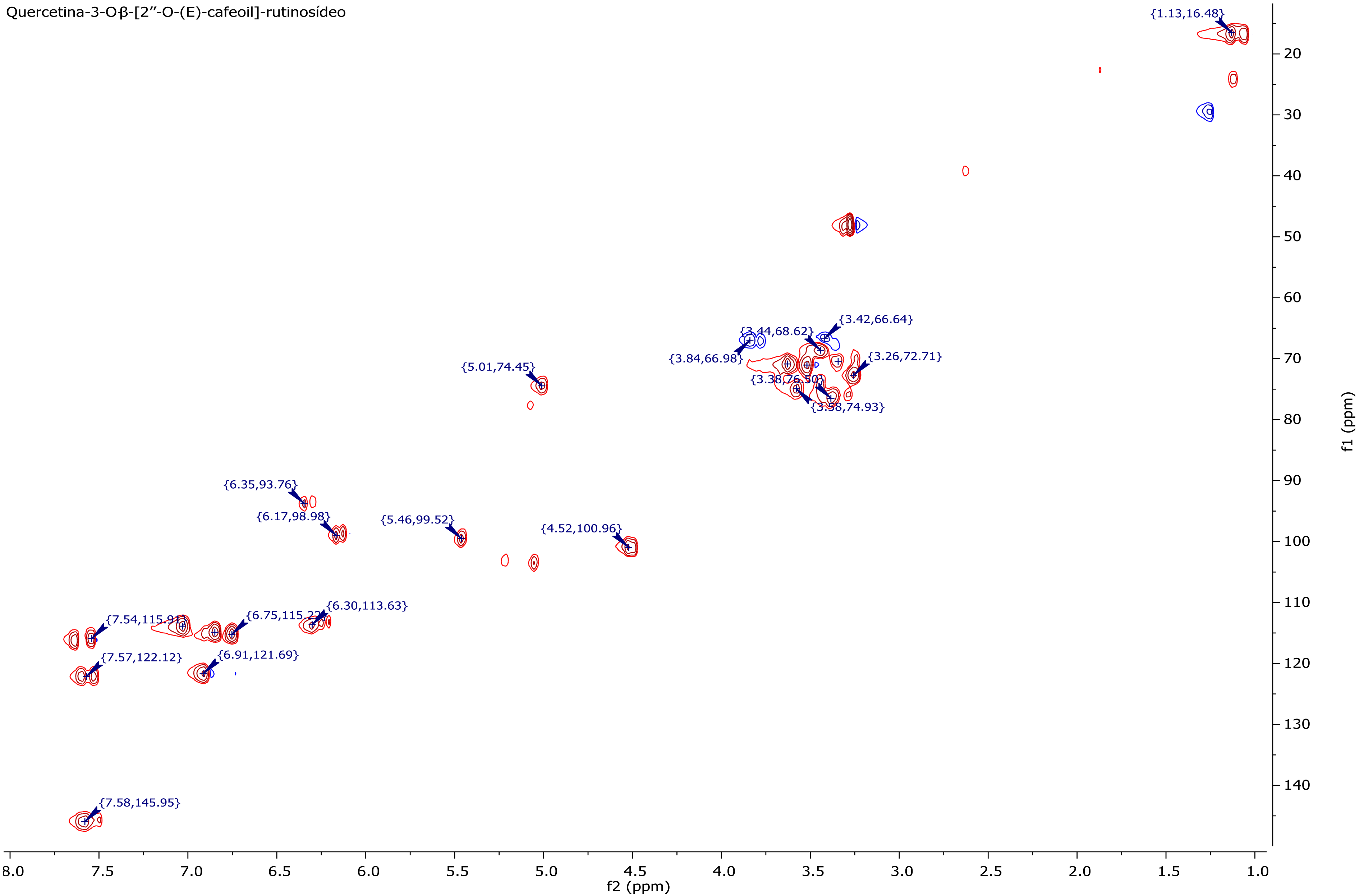

Figure S4. HSQC spectrum of compound 1: oblongifolioside A [quercetin-3-O-β-[2’’-O-(*E*)-caffeoyl]-rutinoside

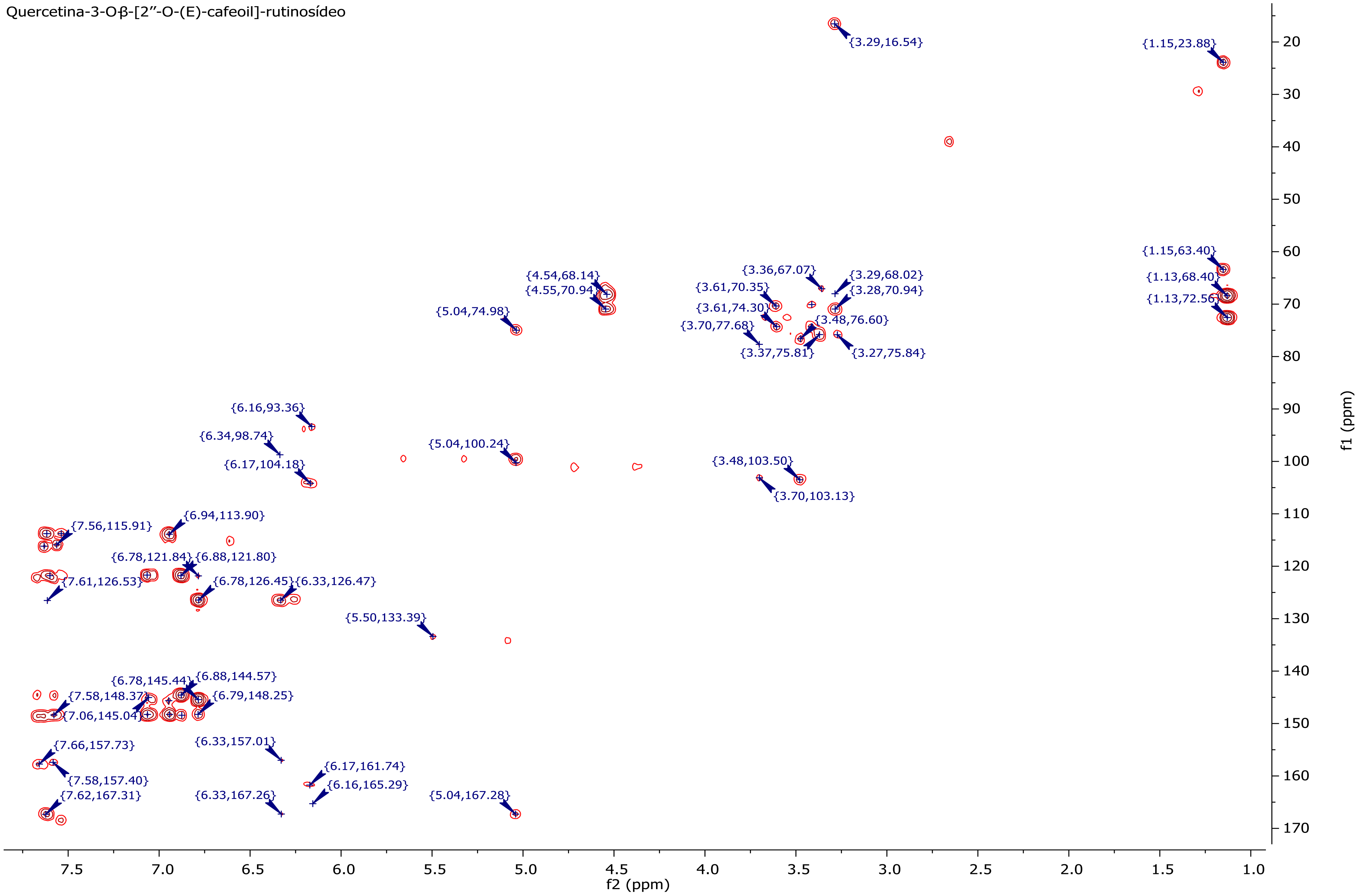

Figure S5. HMBC spectrum of compound 1: oblongifolioside A [quercetin-3-O-β-[2''-O-(*E*)-caffeoyl]-rutinoside

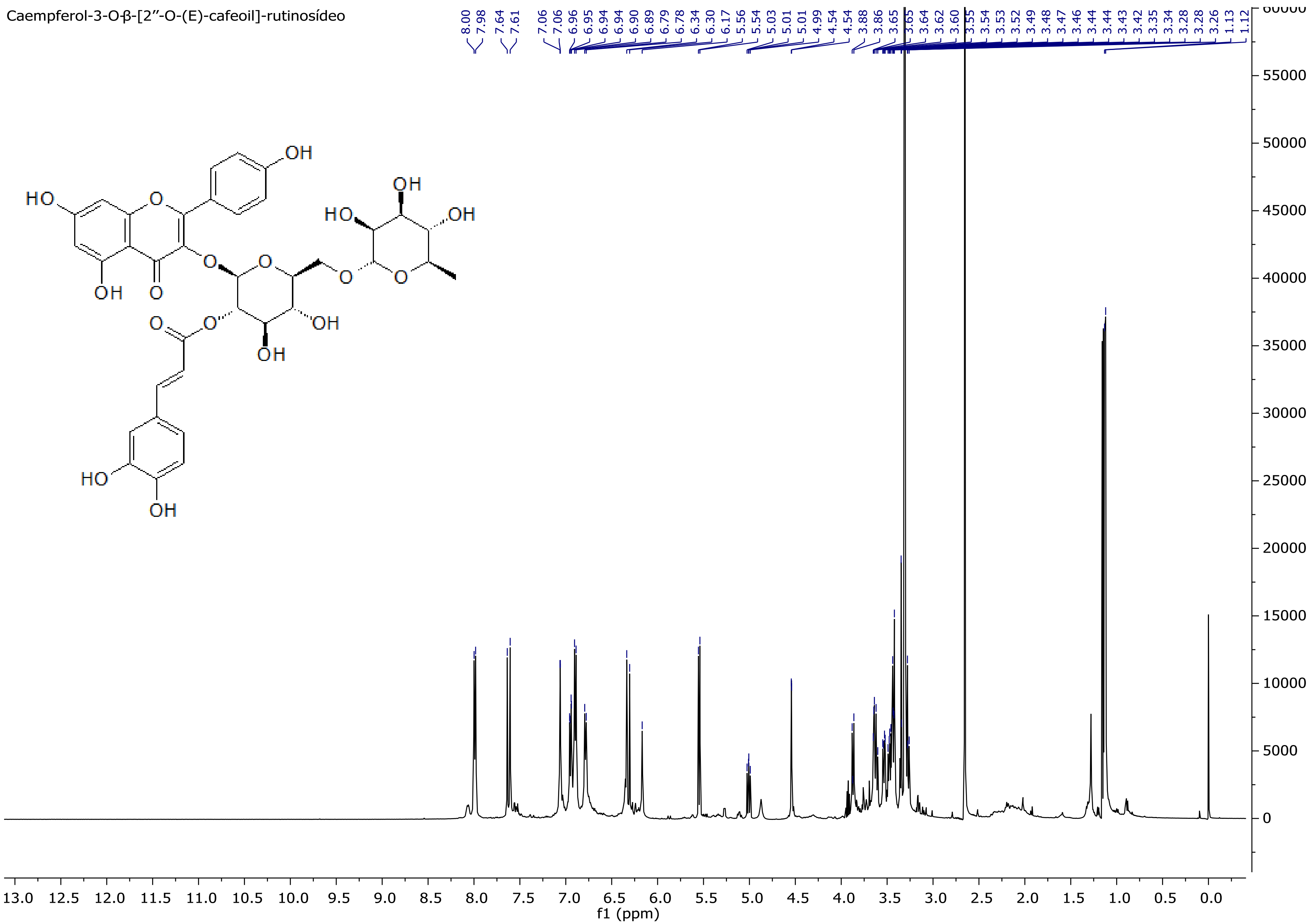

Figure S6. ^{1}H NMR spectrum of compound 2: oblongifolioside B [kaempferol-3-O-β-[2''-O-(*E*)-caffeoyl]-rutinoside

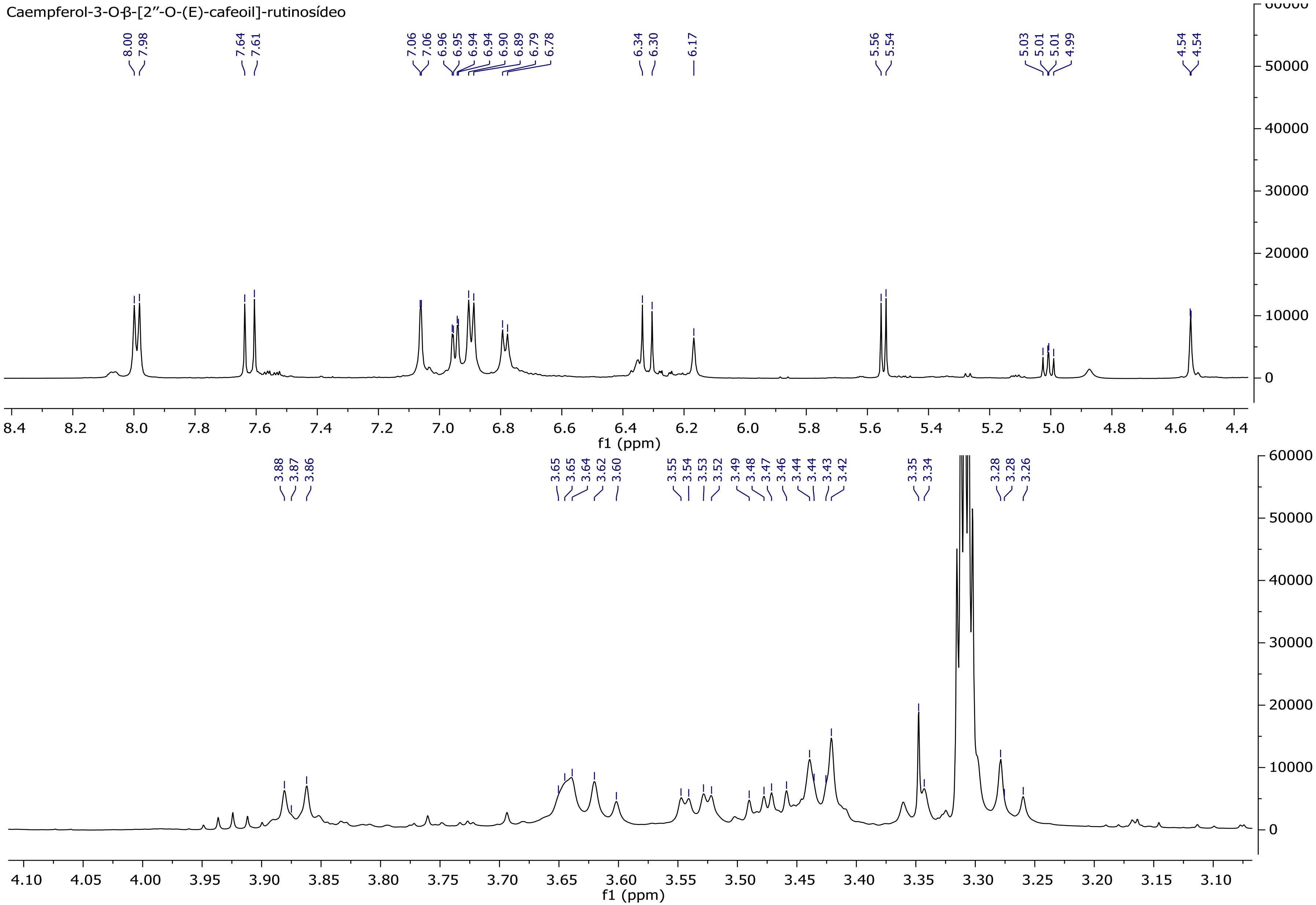

Figure S7. Expansion of ^{1}H NMR of compound 2: oblongifolioside B [kaempferol-3-O-β-[2’’-O-(*E*)-caffeoyl]-rutinoside

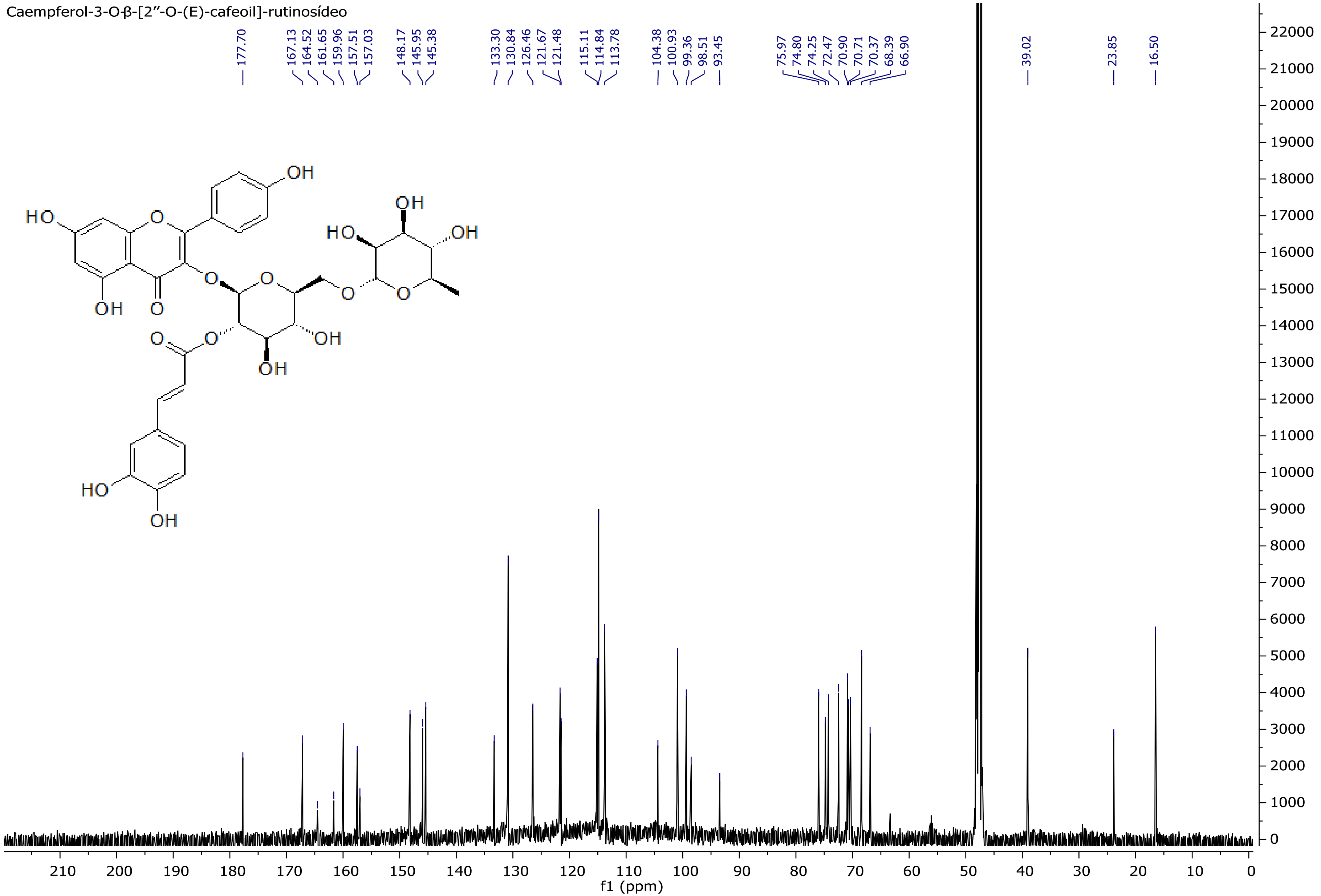

Figure S8. ^{13}C NMR spectrum of compound 2: oblongifolioside B [kaempferol-3-O-β-[2''-O-(*E*)-caffeoyl]-rutinoside

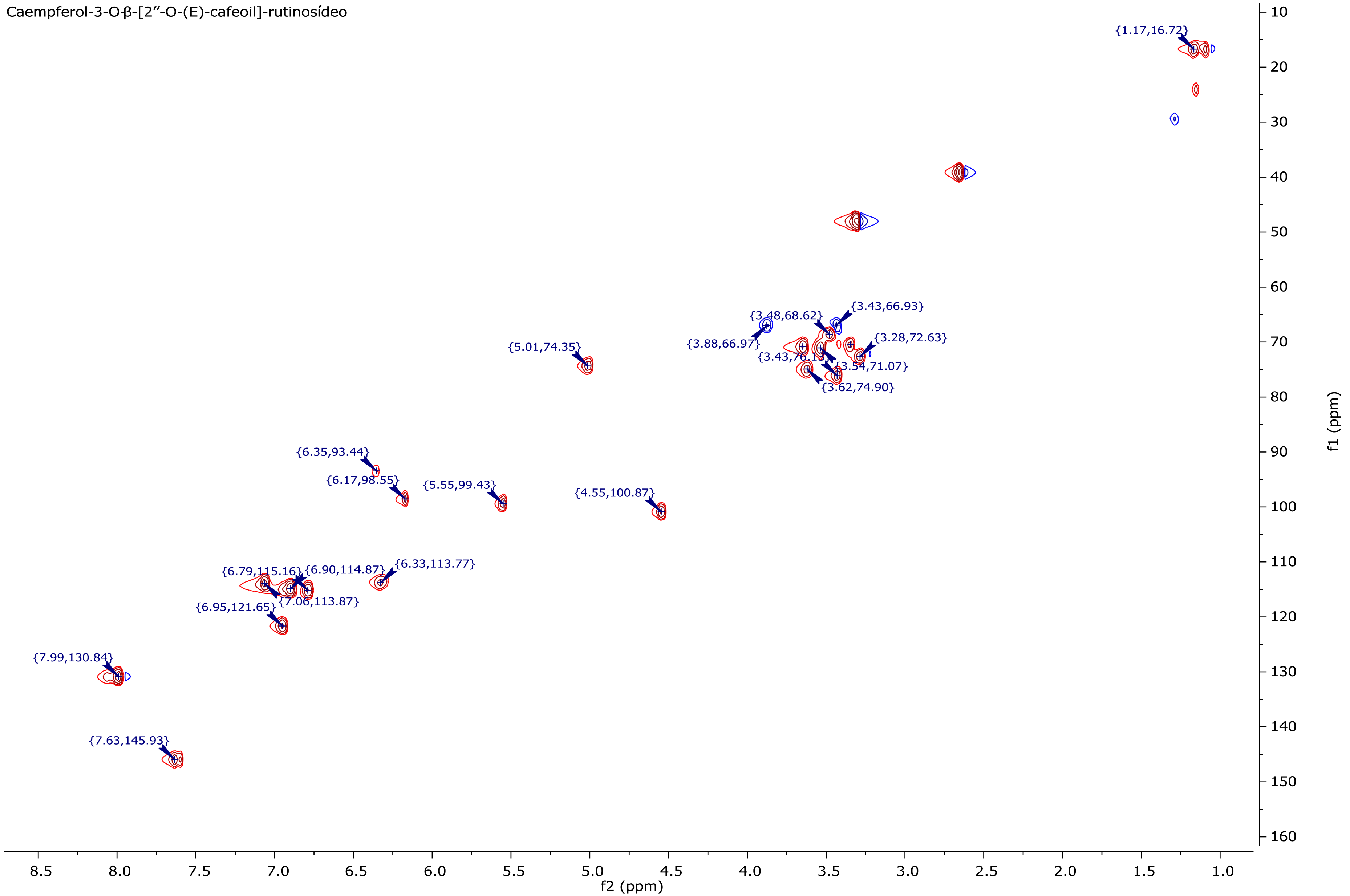

Figure S9. HSQC spectrum of compound 2: oblongifolioside B [kaempferol-3-O-β-[2’’-O-(*E*)-caffeoyl]-rutinoside

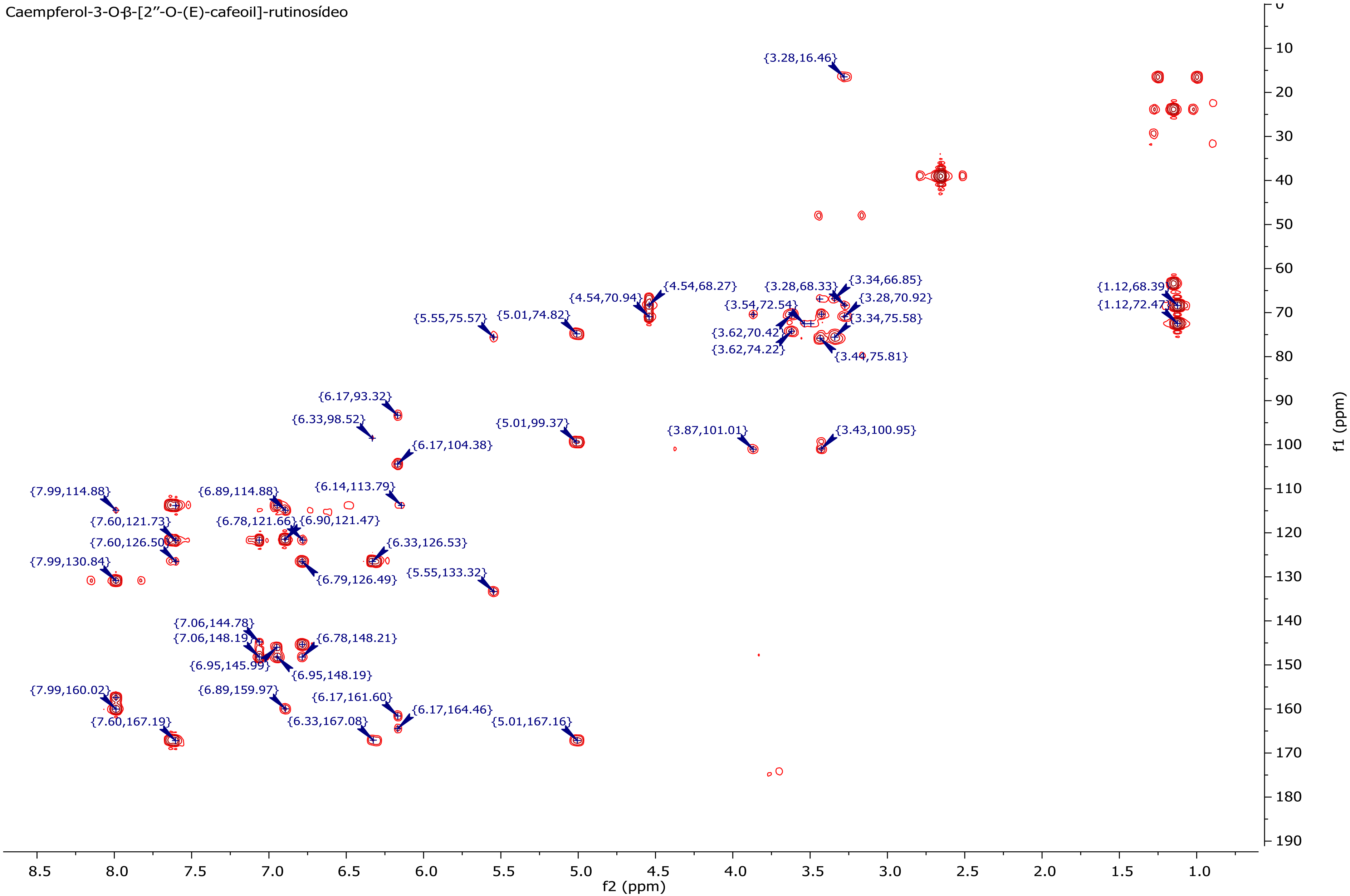

Figure S10. HMBC spectrum of compound 2: oblongifolioside B [kaempferol-3-O-β-[2’’-O-(*E*)-caffeoyl]-rutinoside

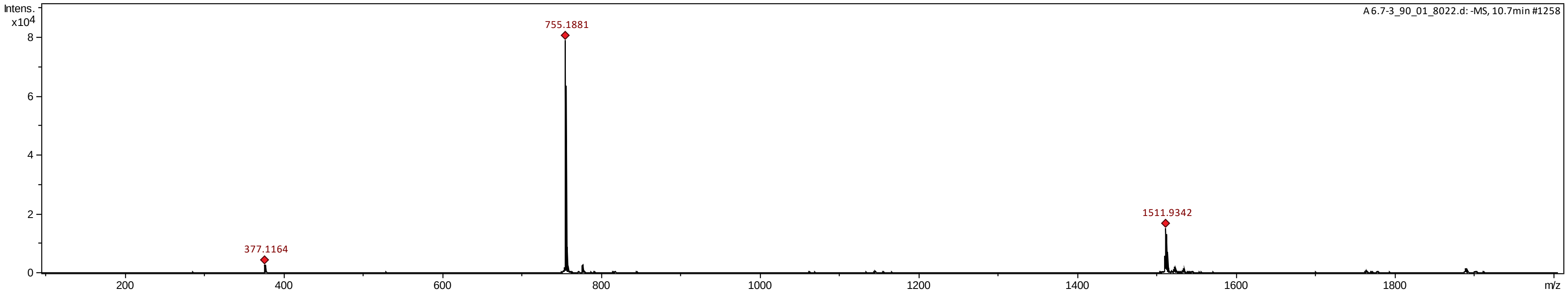

Figure S11. HR-ESI-MS spectrum of compound 2: oblongifolioside B [kaempferol-3-O-β-[2’’-O-(*E*)-caffeoyl]-rutinoside
